# Attitudes of polish medical students toward organ donation

**DOI:** 10.3389/fpubh.2025.1531140

**Published:** 2025-02-03

**Authors:** Marzena Mikla, Anna Maria Cybulska, Antonio Rios, Mariusz Panczyk, Kamila Rachubińska, Artur Kotwas, Beata Karakiewicz, Elżbieta Grochans, Daria Schneider-Matyka

**Affiliations:** ^1^Faculty of Nursing, University of Murcia, Murcia, Spain; ^2^Department of Nursing, Pomeranian Medical University in Szczecin, Szczecin, Poland; ^3^Department of Surgery, Paediatrics, Obstetrics, and Gynaecology, University of Murcia, Murcia, Spain; ^4^Faculty of Health Science, Department of Teaching and Outcomes of Education, Medical University of Warsaw, Warsaw, Poland; ^5^Independent Research and Biostatistics Laboratory, Department of Social Medicine, Pomeranian Medical University in Szczecin, Szczecin, Poland; ^6^Subdepartment of Social Medicine and Public Health, Department of Social Medicine, Pomeranian Medical University in Szczecin, Szczecin, Poland

**Keywords:** brain death, knowledge, medical students, organ donation, transplantation

## Abstract

**Background:**

The aim of the study was to analyze determinants affecting attitudes toward organ donation among medical students at medical universities in Poland.

**Materials and methods:**

The study involved 1,348 medical students. It was performed using a validated questionnaire of attitude toward organ donation and transplantation (ODT) [PCID-DTO RIOS: A questionnaire designed by the International Collaborative Organ Donation project about organ transplantation and donation].

**Results:**

It was shown that those who would not donate their family member’s organs for transplantation were far more likely to believe it was not their moral duty (*p* = 0.013) and to feel no solidarity with those in need (*p* = 0.000). Those who spoke to their families believed it was their moral duty (*p* = 0.000), and believed they would do it out of solidarity with those in need (*p* = 0.000). It was found that having family conversations about donating one’s organs for transplantation was statistically significantly related to being a blood donor (*p* = 0.002), fear of desecration/disfigurement of the body after death in case of organ donation (*p* = 0. 000), a belief that it may be necessary to become an organ recipient in the future (*p* = 0.000), and knowledge of loved ones’ opinions about ODT (father *p* = 0.000, mother *p* = 0.000), partner (*p* = 0.000).

**Conclusion:**

1. The reluctance to donate the organs of loved ones for transplantation is accompanied by a lack of a sense of moral obligation and a lack of solidarity with those in need. Conversely, conversations among loved ones about organ donation are thought-provoking, causing a sense of moral obligation and solidarity with those in need. 2. Regardless of the stance on organ donation and family discussions on the subject, the respondents do not care what happens to the body after organ donation, but they also do not know the opinions of their loved ones about ODT. 3. Conversations with loved ones contribute to the acceptance of circumstances in which organs would be harvested for transplantation without consent.

## 1 Introduction

The word transplantation is derived from the combination of two Latin words: trans—outside and plantare—to plant. By definition, it is a surgical procedure which involves the transfer of an organ or its part, tissue or cell within one or two antigenically compatible organisms (1).

An incurable disease, insufficiency, or irreversible organ damage forces the patient and the therapeutic team to look for effective life-saving methods. The development of transplantology has enabled many patients to continue living (2). In 2018, 140,964 organ transplants were performed worldwide (3). In 2017, the most frequently transplanted organs were kidneys and liver, and the least common were small intestine transplants (4). Organ transplantation is the only recognized treatment for end-stage organ failure. Data from Poltransplant—which is an organizational and coordinating unit for transplantation in Poland—show that every day an average of 15 patients die without receiving a transplant. It is estimated that in 2013 in the European Union (EU) around 4,100 patients on waiting lists died without receiving a transplant (5). The following types of transplants are distinguished by the type of organ donor: transplant from a deceased donor, and transplant from a family donor, i.e., a living donor (the so-called family transplantation). Under current legislation, the procurement of tissue cells and organs from human cadavers is only allowed after the death of the patient has been confirmed, if the deceased did not object to the procurement of their organs during their lives (6). Objection to organ donation can be expressed in three ways: by making an entry in the Central Register of Objections for Deceased Donation, which is housed in the Poltransplant or by objecting to the possible harvesting of one’s own organs after death in writing or orally in the presence of two witnesses (6–8). A declaration of will, which is often associated with consenting directly to harvesting of organs after death, is in fact a document that has no legal force under Polish law. The declaration of will makes it easier to talk to the family of the deceased when the patient is declared dead, but the mere fact of having it is not tantamount to the consent to organ donation after death (9). Once the patient is declared dead, and the committee confirms brain death, the main goal of further care for the potential organ donor is to ensure the proper function of the organs for transplantation. The process of declaring brain death takes place in accordance with the laws in force in Poland, while the deceased can only be considered a potential organ donor after his or her death (7, 10–12). However, in the world there is no uniform definition of a deceased patient, and there are no uniform guidelines to determine brain death (13–15), which causes difficulties in public understanding and acceptance of brain death (16, 17). Whether organs are harvested after death or not is determined by many factors, the first of which is the so-called authorization of organ donation, that is, finding out whether the deceased did not object to organ donation during his or her lifetime. Another aspect that determines organ donation from the deceased is medical factors. From a single deceased organ donor, you can take two kidneys, a heart, a pancreas, a liver, two lungs, an intestine, facial and neck organs, tissues: corneas, fascia, valves, bones and vessels (6, 7, 10, 11, 13, 18).

In the case of a living donor, only one kidney or part of the liver can be transplanted, and then only a child can be the recipient. Under Polish law, a living kidney donor may be a person whose organ is donated to a direct relative, i.e., a sibling, parent, child or spouse with the proviso that donation to another person is possible if it is justified by special personal reasons as stated in Article 13. Pursuant to Article 13, paragraph 1, the procurement of cells, tissues or organs from a living donor for a person who is not a direct relative, a sibling, an adopted person or a spouse requires the consent of the district court with jurisdiction over the donor’s place of residence or stay, issued in non-trial proceedings, after hearing the applicant, and after hearing the opinion of the Ethics Committee of the National Transplantation Council. Article 13 also allows paired organ donation. Organ procurement from a living donor must be preceded by the necessary medical examinations to determine whether the risk of the procedure does not go beyond the limits permissible for this type of procedures and will not significantly affect the donor’s health (6).

The field of transplantation around the world faces a huge disparity between the demand for organs and the possibility of providing them to patients with end-stage organ failure (19, 20). Hence a need to increase donation rates. The shortage of organs, lack of understanding, and general ethical concerns are difficult barriers to organ donation. Overcoming these barriers to reduce imbalance between organ supply and demand requires a multifaceted approach. Key areas include expanding the potential donor pool through educational initiatives and standardized guidelines for both donors and recipients that are needed to increase global awareness and ensure patient safety (21).

In Poland, transplantology is part of the curriculum for medical students. The course covers: issues of surgical transplantation, indications for transplantation of irreversibly damaged organs and tissues, and related procedures as well as the principles of brain death diagnosis (22).

The aim of the study was to identify factors that influence opinions of Polish medical students about ODT.

## 2 Materials and methods

### 2.1 Study population

The research was conducted in 2022 among 1,530 medical students from three medical universities in Poland: the Pomeranian Medical University in Szczecin, Poznań University of Medical Sciences, and the Medical University of Gdańsk.

The study was conducted among students at three medical universities in Poland (Szczecin, Poznan, Gdansk), which allowed for a diverse sample in terms of geographic location and educational contexts. The choice of these specific universities was based on their representativeness for different regions of the country and their importance in medical education.

### 2.2 Sample selection

The study recruited 1,530 medical students from three universities in Poland: the Pomeranian Medical University in Szczecin, Poznań University of Medical Sciences, and the Medical University of Gdańsk.

- 436 respondents from the Pomeranian Medical University in Szczecin, of whom 362 were ultimately included in the study,
- 361 respondents from Poznań University of Medical Sciences, of whom 328 were ultimately included in the study,
- 733 respondents Medical University of Gdańsk, of whom 658 were ultimately included in the study.

The inclusion criteria were: age over 18 years, studying medicine at one of the three medical universities in Poland (Pomeranian Medical University in Szczecin, Poznań University of Medical Sciences, and Medical University of Gdańsk), and giving informed written consent to participate in the study. Ultimately, the study enrolled 1,348 subjects who met all inclusion criteria and correctly completed the questionnaire (completion rate: 88.10%).

Inclusion of adults studying medicine and obtaining written consent to participate in the study ensured compliance with ethical requirements and appropriateness of participants to the study’s purpose.

Prior to the study, approvals were obtained from university authorities and medical departments to conduct the survey at university sites. Surveys were distributed during group classes, lectures, in libraries and other academic spaces. The questionnaires were delivered in paper form. The objectives of the survey and the rules of participation were explained before the questionnaire was filled out. Participants were assured that their responses would be fully anonymous. Respondents were also informed that they could opt out of the survey at any stage. Once completed, the questionnaires were dropped by participants into marked boxes to ensure anonymity.

### 2.3 Ethical issues

This survey-based study was carried out in accordance with the Declaration of Helsinki, and after obtaining the approval from the Bioethics Committee of Pomeranian Medical University (KB-0012/219/06/16). The respondents were informed about the purpose of the study, the possibility of opting out and withdrawing consent at any stage of the study, and were given the opportunity to ask questions and receive comprehensive explanations. The respondents who gave written consent to participate in the study received a questionnaire to fill out.

### 2.4 Instrument for measuring attitude

The study used a standardized questionnaire on attitudes toward ODT [PCID-DTO RIOS: A questionnaire designed by the ‘International Collaborative Organ Donation project about organ transplantation and donation (‘Proyecto Colaborativo Internacional Donante sobre la Donación y Transplante de Organos’ in Spanish) developed by Dr. Ríos) (23). A dependent variable was the attitude toward organ donation, and independent variables were: (1) personal-social, (2) information and knowledge of ODT, (3) social interaction, (4) prosocial behavior, (5) religion, and (6) attitude toward the body. Cronbach’s alpha coefficient for the Spanish population is 0.834. The use of the standardized PCID-DTO RIOS Questionnaire tool made it possible to examine in detail many aspects of attitudes toward ODT in a systematic and reproducible manner.

### 2.5 Statistical analysis

Statistical analysis was performed using the StatisticaTM 13.3 software (TIBCO Software, Palo Alto, CA, USA). Descriptive statistics was used to describe quantitative and categorical variables. Measures determined for the quantitative variables were: central tendency (mean, M) and dispersion (standard deviation, SD), and for the categorical variables—number (N) and frequency (%). Statistical significance was set at *p* < 0.05 (24).

## 3 Results

Women constituted 61.05% and men 38.95% of the study sample. Most of the respondents (45.70%) were aged 21–22 years, 21.36% were under 20 years of age, and less than one-fifth (19.96%) were aged 23–24. The largest group (34.79%) was made up of second-year students, 25.59% were third-year students, and 17.21% were first-year students. Almost half of the respondents (48.81%) were students of Poznań University of Medical Sciences. The majority of the participants (73.29%) lived in a city with a population of over 100,000 inhabitants (Supplementary Table S1). Some 43.47% of the surveyed were practicing Catholics, 28.34% were non-practicing Catholics, while agnostics and atheists accounted for 25.07% (Supplementary Table S2).

### 3.1 Influence of sociodemographic variables on the opinions of polish medical students about ODT

The study analyzed the influence of sociodemographic variables (age, gender, place of residence) and university variables (university, year of study) on the attitudes toward ODT among medical students in Poland.

Analysis of the data showed that the students’ opinions on donating a family member’s organs for transplantation varied depending on where they studied. Students of Poznań University of Medical Sciences were more likely to agree to donate organs of a member of their family for transplantation than their counterparts from other universities. Perhaps the differences in views on ODT are related to curricular differences between the universities. Transplantation learning outcomes can be implemented at different universities at different stages of study. As for the remaining variables (ex. place of residence), no statistically significant differences were found in the context of the respondents’ decision to donate the organs of a family member for transplantation.

When it came to having conversations among relatives about the possibility of donating their organs for transplantation, statistically significant differences by gender were observed. Women talked to their loved ones about ODT more often than men. No statistically significant differences were found for the other variables (Table 1).

**Table 1:** The influence of selected variables on responses to the questions: “Would you donate the organs of your family member for transplantation?” and “Have you discussed transplantation and the possibility of donating your organs for transplantation with your family?”

Variable total: 1348		Would you donate the organs of your family member for transplantation?						Have you discussed transplantation and the possibility of donating your organs for transplantation with your family?					
		No		Yes		*χ* ^2^	*p*	No		Yes		*χ* ^2^	*p*
		*n*	%	*n*	%			*n*	%	*n*	%		
Sex	Male (*n* = 525)	16	47.06	506	38.69	0.978	0.323	271	48.39	252	32.18	36.076	0.000
	Female (*n* = 823)	18	52.94	802	61.31			289	51.61	531	67.82		
Place of residence	Rural area (*n* = 118)	2	5.88	115	8.79	0.365	0.833	52	9.29	66	8.43	3.922	0.141
	Small town (*n* = 242)	6	17.65	233	17.81			113	20.18	128	16.35		
	Big city (*n* = 988)	26	76.47	960	73.39			395	70.54	589	75.22		
Year of study	First (*n* = 232)	4	11.76	227	17.35	2.874	0.719	99	17.68	131	16.73	3.787	0.580
	Second (*n* = 469)	16	47.06	450	34.40			206	36.79	263	33.59		
	Third (*n* = 345)	7	20.59	338	25.84			137	24.46	206	26.31		
	Fourth (*n* = 119)	2	5.88	116	8.87			43	7.68	75	9.58		
	Fifth (*n* = 106)	3	8.82	101	7.72			40	7.14	65	8.30		
	Sixth (*n* = 78)	2	5.88	76	5.81			35	6.25	43	5.49		
University	Pomeranian Medical University in Szczecin (*n* = 363)	14	41.18	347	26.53	7.181	0.028	153	27.32	208	26.56	0.388	0.824
	Medical University of Gdańsk (*n* = 328)	11	32.35	314	24.01			132	23.57	196	25.03		
	Poznań University of Medical Sciences (*n* = 658)	9	26.47	647	49.46			275	49.11	379	48.40		

### 3.2 Influence of attitude-related variables on the opinions of polish medical students about ODT

The willingness to donate a family member’s organs for transplantation differed statistically significantly depending on: being a blood donor (*p* = 0.005), fear of desecration/disfigurement of the body after death in the event of organ donation (*p* = 0.041), and knowledge of the opinions of relatives (father: *p* = 0.003; mother: *p* = 0.000) about ODT. It was shown that 59.10% of the respondents who would donate the organs of a family member for transplantation did not donate blood, but were considering doing so in the future. Half of the respondents who would not donate their loved ones’ organs for transplantation were also considering donating blood in the future. 66.54% of the respondents who would donate the organs of their loved ones for transplantation did not care what happens to the body after death in the event of organ donation. Half of the respondents who answered negatively the question about donating the organs of their loved ones for transplantation were not concerned about the desecration/disfigurement of the body after death in the event of organ donation. 85.29% of the respondents who would not decide to donate their loved ones’ organs for transplantation did not know their fathers’ opinions about ODT, and 61.76% did not know their mothers’ opinions either. The opinion of their father and mother on this subject was not known by 59.62 and 42.91% of the respondents who would donate their loved ones’ organs for transplantation, respectively. Some 73.81% of the respondents who would donate their loved ones’ organs for transplantation and 64.71% of those who would not do so believed that there was a risk that they would become ill and need an organ transplant in the future.

Similarly, a statistically significant relationship was found between being a blood donor (*p* = 0.002), fear of desecration/disfigurement of the body after death in the event of organ donation (*p* = 0.000), a belief that it may be necessary to become an organ recipient in the future (*p* = 0.000), the awareness of loved ones’ opinions on donating organs for transplantation (father’s: *p* = 0.000; mother’s: *p* = 0.000; partner’s: *p* = 0.000), and family discussions about donating organs for transplantation. 59.05% of the respondents whose relatives talked about transplants would like to donate blood in the future. Similarly, 58.33% of those who did not discuss ODT with their relatives were considering donating blood in the future. 73.68% of the respondents who talk to their relatives about ODT did not care what happens to the body after death. 80.32 and 72.45% of the respondents who did not discuss ODT with their relatives did not know the opinions of their fathers or mothers about ODT. 78.42% of the respondents who initiated and 66.67% who did not initiate discussions with their relatives about ODT admitted the risk of having to become an organ recipient in the future (Table 2).

**Table 2:** The influence of selected variables (i.e., being a blood donor, fear of desecration of the body after death in event of organ donation, the possible need to be an organ recipient in the future, and the knowledge of the opinions of the immediate relatives (father, mother, partner) about ODT) on the students’ responses to the questions: “Would you donate the organs of your family member for transplantation?” and “Have you discussed transplantation and the possibility of donating your organs for transplantation with your family?”

Variable		Would you donate the organs of your family member for transplantation?						Have you discussed transplantation and the possibility of donating your organs for transplantation with your family?					
		No		Yes		*χ* ^2^	*p*	No		Yes		*χ* ^2^	*p*
		*n*	%	*n*	%			*n*	%	*n*	%		
Do you donate blood?	Yes, on a regular basis	1	2.94	93	7.18	12.786	0.005	32	5.80	62	7.96	14.768	0.002
	Yes, sometimes or only once	5	14.71	280	21.60			107	19.38	179	22.98		
	No, but I’d like to	17	50.00	766	59.10			322	58.33	460	59.05		
	No and I’m not going to	11	32.35	157	12.11			91	16.49	78	10.01		
Are you afraid that if you became an organ donor, your body would be desecrated and disfigured after death?	Yes, I do	8	23.53	141	10.82	6.386	0.041	80	14.31	70	8.99	45.721	0.000
	No, I do not care	17	50.00	867	66.54			313	55.99	574	73.68		
	I do not know	9	26.47	295	22.64			166	29.70	135	17.33		
Do you know your father’s opinion about ODT?	Yes, he is for	2	5.88	447	34.25	14.320	0.003	87	15.56	360	46.09	165.520	0.000
	Yes, he is against	3	8.82	51	3.91			11	1.97	44	5.63		
	I do not know	29	85.29	778	59.62			449	80.32	359	45.97		
	Other	0	0.00	29	2.22			12	2.15	18	2.30		
Do you know your mother’s opinion about ODT?	Yes, she is for	3	8.82	631	48.35	38.017	0.000	127	22.72	505	64.66	328.790	0.000
	Yes, she is against	10	29.41	87	6.67			21	3.76	77	9.86		
	I do not know	21	61.76	560	42.91			405	72.45	177	22.66		
	Other	0	0.00	27	2.07			6	1.07	22	2.82		
If you are in a relationship, do you know your partner’s opinion about ODT?	Yes, he/she is for	6	18.18	413	31.89	4.051	0.256	127	23.13	293	37.61	93.961	0.000
	Yes, he/she is against	2	6.06	32	2.47			11	2.00	23	2.95		
	I do not know	8	24.24	263	20.31			179	32.60	91	11.68		
	I’m single	17	51.52	587	45.33			232	42.26	372	47.75		
Do you believe you will ever need an organ for transplantation?	Yes, it is possible that I’ll get sick	22	64.71	964	73.81	2.110	0.348	372	66.67	614	78.42	23.197	0.000
	No, I have a healthy lifestyle	3	8.82	58	4.44			33	5.91	28	3.58		
	I do not know	9	26.47	284	21.75			153	27.42	141	18.01		

*Pearson’s chi-square test.

Discussions with family members about donating their organs for transplantation were statistically significantly related to religion (*p* = 0.001), and opinions about harvesting organs from a deceased person without consent (*p* = 0.000). 47.65% of the practicing Catholics did not discuss and 41.42% discussed ODT with their relatives. 52.33% of the respondents who had family talks about ODT believed that harvesting organs without prior consent would be a good way to use them. No statistically significant relationships were found between the other variables and family discussions about donating organs for transplantation. There were also no statistically significant associations between the variables from Table 3 and the willingness to donate a family member’s organs for transplantation (Table 3).

**Table 3:** The influence of selected variables (i.e., religion, the opinion of one’s church on ODT, and a law that would allow state institutions to take the organs of the deceased without prior permission) on responses to the questions: “Would you donate the organs of your family member for transplantation?” and “Have you discussed transplantation and the possibility of donating your organs for transplantation with your family?”

Variable		Would you donate the organs of your family member for transplantation?						Have you discussed transplantation and the possibility of donating your organs for transplantation with your family?					
		No		Yes		*χ* ^2^	*p*	No		Yes		*χ* ^2^	*p*
		*n*	%	*n*	%			*n*	%	*n*	%		
Are you…?	Agnostic, atheist	7	21.21	327	25.25	1.876	0.599	109	19.68	227	29.29	16.262	0.001
	Practicing Catholic	18	54.55	566	43.71			264	47.65	321	41.42		
	Non-practicing Catholic	7	21.21	375	28.96			167	30.14	213	27.48		
	Another religion	1	3.03	27	2.08			14	2.53	14	1.81		
What, do you think, is the attitude of the church you belong to toward ODT?	It is for	20	64.52	779	65.35	1.989	0.370	338	64.38	461	66.05	0.461	0.794
	It is against	1	3.23	116	9.73			53	10.10	64	9.17		
	I do not know	10	32.26	297	24.92			134	25.52	173	24.79		
What would you say about a law that would allow state institutions to take the organs of the deceased without prior permission?	A wonderful gesture of solidarity	4	12.12	106	8.19	3.914	0.271	50	9.03	60	7.75	34.590	0.000
	A good use of organs	12	36.36	611	47.22			216	38.99	405	52.33		
	The authority has exceeded its powers	13	39.39	506	39.10			239	43.14	284	36.69		
	For the family, it is like a slap in the face	4	12.12	71	5.49			49	8.84	25	3.23		

*Pearson’s chi-square test.

### 3.3 Influence of information sources on the attitudes of polish medical students toward ODT

The sources of information about ODT were found to have an impact on the respondents’ willingness to donate their family members’ organs for transplantation, and on whether they had family discussions about organ donation—the differences were statistically significant. In the case of family discussions, important sources of information were: TV (*p* = 0.001), books (*p* = 0.003), magazines (*p* = 0.017), friends (*p* = 0.000), family (*p* = 0.000), movies (*p* = 0.000), doctors and nurses (*p* = 0.012), school (*p* = 0.000), internet (*p* = 0.002), social media (*p* = 0.000) and church (*p* = 0.001) (Supplementary Table S3).

Based on the data, those respondents who would not donate the organs of their family members for transplantation were far more likely to believe that it was not their moral duty (*p* = 0.013), and did not feel solidarity with those in need (*p* = 0.000).

It was also noted that those who talked to family members about the possibility of donating their organs in the future believed it was their moral duty (*p* = 0.000), and claimed that they would do so out of solidarity with people in need (*p* = 0.000). At the same time, those respondents would not want to survive their own death (*p* = 0.038), and would not agree to an organ transplant for religious reasons (*p* = 0.028) (Table 4).

**Table 4:** The influence of selected variables on responses to the questions: “Would you donate the organs of your family member for transplantation?” and “Have you discussed transplantation and the possibility of donating your organs for transplantation with your family?”

Variable		Would you donate the organs of your family member for transplantation?						Have you discussed transplantation and the possibility of donating your organs for transplantation with your family?					
		No		Yes		*χ* ^2^	*p*	No		Yes		*χ* ^2^	*p*
		*n*	%	*n*	%			*n*	%	*n*	%		
Because I think it is my moral duty	Yes	10	29.41	668	51.07	6.219	0.013	240	42.86	437	55.81	21.916	0.000
	No	24	70.59	640	48.93			320	57.14	346	44.19		
Because of solidarity with those in need	Yes	13	38.24	892	68.20	13.546	0.000	345	61.61	558	71.26	13.823	0.000
	No	21	61.76	416	31.80			215	38.39	225	28.74		
Because I want to survive my own death	Yes	2	5.88	40	3.06	0.872	0.350	11	1.96	31	3.96	4.289	0.038
	No	32	94.12	1,268	96.94			549	98.04	752	96.04		
For religious reasons	Yes	2	5.88	66	5.05	0.048	0.826	20	3.57	49	6.26	4.835	0.028
	No	32	94.12	1,242	94.95			540	96.43	734	93.74		
Because it is free	Yes	3	8.82	91	6.96	0.177	0.674	44	7.86	51	6.51	0.897	0.344
	No	31	91.18	1,217	93.04			516	92.14	732	93.49		
Because I expect someone else to do the same for me in the future	Yes	8	23.53	502	38.38	3.102	0.078	204	36.43	307	39.21	1.070	0.301
	No	26	76.47	806	61.62			356	63.57	476	60.79		
Other reasons	Yes	3	8.82	155	11.85	0.292	0.589	59	10.54	99	12.64	1.398	0.237
	No	31	91.18	1,153	88.15			501	89.46	684	87.36		

*Pearson’s chi-square test.

## 4 Discussion

Medical advances over the past decades have made organ transplantation the ultimate treatment for end-stage organ failure. However, unavailability of organs needed for transplantation is a major challenge worldwide.

Our research shows that regardless of whether there are discussions in the respondents’ families about donating organs for transplantation, the respondents do not know the opinions of their relatives on this issue. What is more, most respondents who would not consent to donating their loved ones’ organs for transplantation do not know their opinion on this matter. Costa-Front et al., who used data from 28 European countries for 2002–2010, found that presumed consent policies were associated with increased willingness to donate organs, but this effect was attenuated when internal family discussions about ODT were taken into account. It has been established that the consent of a relative acts as a veto on the intention to donate organs for transplantation and weakens the influence of legal regulations on actual donations. Presumed consent increases the willingness to donate one’s own and relatives’ organs for transplantation in countries where family consent is not required. This effect is due to the impact of family discussions, which increased the willingness to donate organs for transplantation and, combined with presumed consent, have translated into higher ODT rates (25).

Our study shows a similar relationship between family conversations and positive attitudes toward ODT, highlighting the importance of these conversations. However, the difference in results regarding the impact of implicit consent (no need for family consent) may be due to cultural and legal differences between Poland and the countries studied. In the study by Symvoulakis et al. about one-third of respondents (34.0%) discussed the presumed consent with a partner, family member or friend. More than half (54.2%) were afraid that donated organs could be used without consent for other purposes, such as medical research (26). This is not supported by our research, which shows that respondents who discussed ODT with their families would accept the situation of using organs for transplantation without permission. In the study group, it also did not matter what happens to the body after organ donation, regardless of views on ODT. The difference may be due to differences in the level of trust in the health care system or the lack of clear regulations. It is worth looking at how other countries are addressing this challenge, such as through trust-building education campaigns.

Our research has confirmed the influence of gender and religion on the initiation of discussions about donating organs for transplantation among immediate family members.

In the study by Guo et al., out of 4,565 people surveyed, 621 clearly stated that they did not consent to organ donation after death, 701 people expressed their willingness to donate organs after death, but only 259 respondents signed the declaration of will. The willingness of respondents to donate organs statistically significantly differed by gender, age, religious beliefs, place of residence, and level of education (27). In the UK, on the other hand, the patient’s ethnicity, religious beliefs, gender, and socio-economic status, as well as the knowledge of ODT were strongly associated with consent to donate organs (*p* < 0.001) (28). According to Umar et al., religiousness was a factor strengthening the relationship between the willingness to donate organs and signing a donor card (29).

The results of Guo et al. (27) and Umar et al. (29) indicate that gender, age, religious beliefs, place of residence and education have a significant effect on willingness to donate organs. Our results are consistent on the influence of gender and religiosity, but the differences are related to place of residence. This may suggest that in Poland the influence of local communities and cultural traditions is smaller compared to other countries. It would be worthwhile to analyze how different demographic factors may shape attitudes in the Polish context.

In the study by Symvoulakis et al., 30% of respondents considered organ donation unacceptable due to religious beliefs [28]. A meta-analysis concerning the knowledge of ODT and willingness to donate organs showed that they vary by population and location. In fact, they are linked to a number of social factors, and ultimately lead to the consent of potential donors to have their organs harvested for transplantation. Family influence, religious, traditional and spiritual beliefs, as well as the status of ethnic, minority or immigrant group are important determinants of the decision to donate organs (30).

Our research indicates that respondents who would not consent to donating their family member’s organs for transplantation believe that it is not their moral duty and do not feel solidarity with those in need. Conversations with family members about the possibility of donating their organs in the future provoked reflection and contributed to feelings of moral obligation and solidarity with the recipients.

At Shahid Beheshti Medical University, perceived behavioral control was correlated with the students’ intention to receive an organ donor card. The authors believe that facilities should be provided so that students can easily register as organ donors (31).

The perception of behavioral control, moral norms, and attitudes are significant predictors of the willingness to donate organs after death. It has also been shown that those who were willing to sign a donor card were also ready to communicate their decision to their families (28).

The results of our study indicate that family conversations stimulate moral reflection and foster a sense of solidarity with those in need. This correlates with studies in the UK and at Shahid Beheshti University (28, 31), where moral attitudes, social norms and perceptions of behavioral control influence decisions about organ donation. It can be suggested that educational campaigns in Poland should place more emphasis on the ethical aspects of organ donation, which could effectively increase the number of donors.

Study Akbulut et al. (32) indicates that medical students have inadequate knowledge, attitudes, and behaviors about organ donation. In the study by Fan et al. (33), knowledge and attitudes were found to be positively related to the Chinese public’s willingness to donate organs. The knowledge of brain death issues and the transplant procedure can help increase the willingness to donate organs. Therefore, it is crucial to understand social determinants of ODT, and this aspect should be addressed when developing policies for public awareness and educational initiatives. Akbulut et al. (32) and Fan et al. (33), the level of knowledge and understanding of transplantation procedures were shown to be significant determinants of willingness to donate organs. Similarly, in our study, lack of knowledge of family opinions correlates with lower willingness to consent to organ donation. It is worth discussing how educational programs in Poland can be adapted to increase public awareness and medical knowledge, especially among students.

### 4.1 Practical implications

This research of students’ opinions on organ donation, due to differences in results between students at different universities, highlights the need to standardize educational content on transplantation in college curricula. Introducing uniform, well-designed educational modules on organ donation could increase students’ awareness and positive attitudes. Since family conversations are crucial in forming opinions on organ donation, public campaigns should encourage young people to initiate such discussions. Workshops and educational events could be considered to help students gain the knowledge and tools to have conversations with loved ones. The results of our own research indicate that women are more likely to have conversations about organ donation with relatives, suggesting the need for gender-specific communication strategies. Awareness campaigns can direct special attention to engaging men in conversations about organ donation. In turn, the link between being a blood donor and willingness to donate organs suggests that programs that encourage blood donation may also influence attitudes toward organ donation. Initiatives to encourage blood donation can be expanded to include education about transplantation.

This study also indicates that various sources of information, such as social media, movies, schools and churches, had a significant impact on students’ attitudes toward donation. Campaigns promoting ODT should use a variety of communication channels, tailored to young people’s preferences, to increase their involvement and awareness. Given the influence of the media and the Internet on students’ attitudes, it is worthwhile to ensure the availability of credible and easily understood materials on ODT in these channels. The results of this study also suggest that it is worth including ethical, moral and religious contexts in educational campaigns, as beliefs strongly influence attitudes toward organ donation. Including religious leaders could support the promotion of knowledge regarding the compatibility of organ donation with different belief systems.

Importantly, campaigns promoting ODT should emphasize the importance of solidarity with those in need of transplants and the moral obligation to help others. Including emotional narratives about people who have benefited from transplants can be effective in building a sense of community.

The findings point to the need for further monitoring and analysis of attitudes toward ODT in different demographic groups. Regular surveys could help adjust educational and promotional strategies to meet changing social needs. Implementation of these measures could help increase acceptance and understanding of organ donation in Poland, which would ultimately improve the availability of organs for transplantation.

## 5 Conclusion

1. Women were more likely than men to discuss donating organs for transplantation with their immediate family members.
2. The reluctance to donate the organs of loved ones for transplantation is accompanied by a lack of a sense of moral obligation and a lack of solidarity with those in need. Conversely, conversations among loved ones about ODT are thought-provoking, causing a sense of moral obligation and solidarity with those in need.
3. Regardless of the stance on ODT and family discussions on the subject, the respondents do not care what happens to the body after organ donation, but they also do not know the opinions of their loved ones about ODT.
4. Conversations with loved ones contribute to the acceptance of circumstances in which organs would be harvested for transplantation without consent.

### 5.1 Limitation

This study has some limitations. First, the cross-sectional design of this study limits the ability to draw definite conclusions about the causal relationship. Second, the results do not allow generalized conclusions about all medical university students at home or abroad. The study sample should be expanded to include students from other medical universities. Additionally, the analysis should take into account curricular differences regarding brain death issues.

The PCID-DTO RIOS questionnaire, while a comprehensive tool, did not address all contextual aspects, such as initiating family discussions, reasons for gender differences, or the impact of regional cultural differences. Expand future research by supplementing the tool with questions exploring these issues and using qualitative methods to obtain more in-depth data. Expanding the analysis in these areas will allow for a more comprehensive understanding of attitudes toward organ donation in the Polish context.

## Data Availability

The raw data supporting the conclusions of this article will be made available by the authors, without undue reservation.

## References

[1] Makara-Studzińska M Kowalska AJ Jakubowska K. Poziom wiedzy oraz opinie pielęgniarek na temat transplantacji organów. Med Og Nauk Zdr. (2012) 18:31–6.

[2] Picewicz J. Zagadnienie transplantacji w dokumentach społecznej nauki Kościoła. Rocz Filoz Ignat. (2015) 1:98–114.

[3] Global Observatory on Donation and Transplantation. Summary. (2018). Available at: http://www.transplant-observatory.org/summary/

[4] Global Observatory on Donation and Transplantation. International report on organ donation and transplantation activities. (2017). Available at: http://www.transplant-observatory.org/download/2017-activity-data-report/

[5] European Commission. Journalist workshop on organ donation and transplantation: Recent facts and figures. Available at: http://ec.europa.eu/health/blood_tissues_organs/docs/ev_20141126_factsfigures_en.pdf (Accessed October 31, 2023).

[6] Ustawa z dnia 1 lipca 2005 r. o pobieraniu, przechowywaniu i przeszczepianiu komórek, tkanek i narządów. Dz.U. 2005 nr 169 poz. 1411. ISAP.

[7] Obwieszczenie Ministra Zdrowia z dnia 4 grudnia 2019 r. w sprawie sposobu i kryteriów stwierdzenia trwałego nieodwracalnego ustania czynności mózgu. M.P. 2020 poz. 73. Dziennik Ustaw.

[8] Biuletyn Centrum Organizacyjno-Koordynacyjnego ds. Transplantacji “Poltransplant”. (2022). Available at: www.Poltransplant.com (Accessed November 14, 2022).

[9] Skalski J. Historia przeszczepiania narządów. CX News. (2009) 2:20–25.

[10] Guzik-Makaruk EM. Transplantacje organów, tkanek i komórek w ujęciu prawnym i kryminologicznym. Białystok: Temida 2, (2008).

[11] Giezek J Kokot R Banaszak B Preisner A. Granice ludzkiego życia a jego prawna ochrona. Prawa i wolności obywatelskie w Konstytucji RP. eds. Banaszak B. Preisner A. (Warszawa: Wydawnictwo Sejmowe.) (2002) 99–117.

[12] Duda J. Transplantacja w prawie polskim: aspekty cywilnoprawne. Kraków: Aspekty cywilnoprawne (1998).

[13] Da Silva IRF Frontera JA. Worldwide barriers to organ donation. JAMA Neurol. (2015) 72:112–8. doi: 10.1001/jamaneurol.2014.3083, PMID: 25402335

[14] Freeman RB Bernat JL. Ethical issues in organ transplantation. Prog Cardiovasc Dis. (2012) 55:282–9. doi: 10.1016/j.pcad.2012.08.005, PMID: 23217432

[15] Jones DA. Loss of faith in brain death: Catholic controversy over the determination of death by neurological criteria. Clin Ethics. (2012) 7:133–41. doi: 10.1258/ce.2012.012m07

[16] Siminoff LA Burant C Youngner SJ. Death and organ procurement: public beliefs and attitudes. Soc Sci Med. (2004) 59:2325–34. doi: 10.1016/j.socscimed.2004.03.029, PMID: 15450707

[17] Kirby J. Organ donation after assisted death: is it more or less ethically problematic than donation after circulatory death? Med Health Care Philos. (2016) 19:629–35. doi: 10.1007/s11019-016-9711-8, PMID: 27263089

[18] Biesaga T. Wobec zgody domniemanej i zawłaszczania zwłok do transplantacji. Med Pr. (2006) 1:20–24.

[19] Nowacka M Szewczyk K. Pacjent w systemie opieki zdrowotnej. Warszawa: Wydawnictwo Lekarskie PZWL (2009).

[20] Kubicki L. Aktualny stan prawny w odniesieniu do pobierania i przeszczepiania komórek, tkanek i narządów. W: Raport o stanie przeszczepiania komórek, tkanek i narządów w Polsce. Warszawa: Centrum Organizacyjno-Koordynacyjne ds. Transplantacji “Poltransplant”. (2000) 6.

[21] Lewis A Koukoura A Tsianos GI Gargavanis AA Nielsen AA Vassiliadis E. Organ donation in the US and Europe: the supply vs demand imbalance. Transpl Rev. (2021) 35:100585. doi: 10.1016/j.trre.2020.100585, PMID: 33071161

[22] Pomeranian Medical University in Szczecin. Available at: https://www.pum.edu.pl/studia_iii_stopnia/student/wydzia_medycyny_i_stomatologii/kierunek_lekarski/plany_studiow_lekarski/ (Accessed April 26, 2024).

[23] Ríos A López-Navas AI De-Francisco C Sánchez Á Hernández AM Ramírez P. Psychometric characteristics of the attitude questionnaire toward the donation of organs for transplant (PCID-DTO-RIOS). Transpl Proc. (2018) 50:345–9. doi: 10.1016/j.transproceed.2017.11.063, PMID: 29579800

[24] Mishra P Pandey CM Singh U Keshri AU Sabaretnam M. Selection of appropriate statistical methods for data analysis. Ann Card Anaesth. (2019) 22:297–301. doi: 10.4103/aca.ACA_248_18, PMID: 31274493 PMC6639881

[25] Costa-Font J Rudisill C Salcher-Konrad M. ‘Relative consent’ or ‘presumed consent’? Organ donation attitudes and behaviour. Eur J Health Econ. (2021) 22:5–16. doi: 10.1007/s10198-020-01214-8, PMID: 32651683 PMC7822792

[26] Symvoulakis E Markaki A Rachiotis G Linardakis M Klinis S Morgan M. Organ donation attitudes and general self-efficacy: exploratory views from a rural primary care setting. Rural Remote Health. (2019) 19(4):[1]–[8]. doi: 10.22605/RRH5241, PMID: 31661290

[27] Guo H Hou X Ren J Cui Q Wang X Yu X. Analysis of influencing factors of organ donation willingness based on ABC attitude model. Organ Transpl. (2022):378–8.

[28] Curtis RMK Manara AR Madden S Brown C Duncalf S Harvey D. Validation of the factors influencing family consent for organ donation in the UK. Anaesthesia. (2021) 76:1625–34. doi: 10.1111/anae.15485, PMID: 33860929

[29] Umair S Ho JA Ng SSI Basha NK. Moderating role of religiosity and the determinants to attitude, willingness to donate and willingness to communicate posthumous organ donation decisions among university students in Pakistan. Omega J Death Dying. (2023) 88:216–44. doi: 10.1177/00302228211045170, PMID: 34505539

[30] Mekkodathil A El-Menyar A Sathian B Singh R Al-Thani H. Knowledge and willingness for organ donation in the middle eastern region: a Meta-analysis. J Relig Health. (2020) 59:1810–23. doi: 10.1007/s10943-019-00883-x, PMID: 31309441 PMC7359145

[31] Ghaffari M Rakhshanderou S Najafizadeh K Ramezankhani A Latifi M. Determinants of medical students for intention to organ donation: application of theory of planned behavior. Saudi J Kidney Dis Transpl. (2019) 30:1375–80. doi: 10.4103/1319-2442.275481, PMID: 31929284

[32] Akbulut S Demyati K Toman I Gaygili Z Kaya S Akpolat VR. Medical students’ knowledge, attitudes, and awareness toward organ donation. Transpl Immunol. (2022) 73:101634. doi: 10.1016/j.trim.2022.101634, PMID: 35623595

[33] Fan X Li M Rolker H Li Y Du J Wang D. Knowledge, attitudes, and willingness to organ donation among the general public: a cross-sectional survey in China. BMC Public Health. (2022) 22. doi: 10.1186/s12889-022-13173-1, PMID: 35534843 PMC9082919

