# Supplementary material for: Attitudes of polish medical students toward organ donation
Source: Front Public Health. 2025 Feb 3;13:1531140. doi: 10.3389/fpubh.2025.1531140 (PMC11831047; doi:10.3389/fpubh.2025.1531140)
Supplement: Supplementary file 1 [file Table_1.DOCX]

Table S1. Characteristics of the study group (n = 1348).

|  | N | % |
| --- | --- | --- |
| Gender |  |  |
| W | 823 | 61.05 |
| M | 525 | 38.95 |
| Age |  |  |
| ≤20 | 288 | 21.36 |
| 21-22 | 616 | 45.70 |
| 23-24 | 269 | 19.96 |
| 25-26 | 125 | 9.27 |
| 27-28 | 31 | 2.30 |
| >28 | 19 | 1.41 |
| Year of study |  |  |
| 1 | 232 | 17.21 |
| 2 | 469 | 34.79 |
| 3 | 345 | 25.59 |
| 4 | 119 | 8.83 |
| 5 | 105 | 7.79 |
| 6 | 78 | 5.79 |
| University |  |  |
| PUM | 362 | 26.85 |
| GUM | 328 | 24.33 |
| UMP | 658 | 48.81 |
| Place of residence |  |  |
| village | 118 | 8.75 |
| city 10-100 thousand | 242 | 17.95 |
| city >100 thousand | 988 | 73.29 |

Table S2. Characteristics regarding the religion of the study group.

| Confession | N | % |
| --- | --- | --- |
| Practicing Catholic | 586 | 43.47 |
| Non-practicing Catholic | 382 | 28.34 |
| Agnostic, atheist | 338 | 25.07 |
| Other Roman Catholic Church | 12 | 0.89 |
| Protestant | 8 | 0.59 |
| Judaism | 5 | 0.37 |
| Orthodox | 3 | 0.22 |
| No answer | 14 | 1.04 |

Table S3. Sources of opinions regarding organ transplantation

| Sources of opinions regarding organ transplantation |  | | | | | | | |
| --- | --- | --- | --- | --- | --- | --- | --- | --- |
|  | Yes, positive | | yes, negative | | No | | no answer | |
|  | n | % | n | % | n | % | n | % |
| TV | 901 | 66.84 | 33 | 2.45 | 385 | 28.56 | 29 | 2.15 |
| Radio | 347 | 25.74 | 20 | 1.48 | 900 | 66.77 | 81 | 6.01 |
| Books, brochures | 960 | 71.22 | 16 | 1.19 | 329 | 24.41 | 43 | 3.19 |
| Magazines, newspapers | 654 | 48.52 | 40 | 2.97 | 590 | 43.77 | 64 | 4.75 |
| Movies | 786 | 58.31 | 60 | 4.45 | 440 | 32.64 | 62 | 4.60 |
| Friends | 893 | 66.25 | 48 | 3.56 | 353 | 26.19 | 54 | 4.01 |
| Family | 610 | 45.25 | 82 | 6.08 | 589 | 43.69 | 67 | 4.97 |
| Billboards, boards | 578 | 42.88 | 17 | 1.26 | 690 | 51.19 | 63 | 4.67 |
| Doctors, nurses | 807 | 59.87 | 7 | 0.52 | 491 | 36.42 | 43 | 3.19 |
| School | 925 | 68.62 | 19 | 1.41 | 368 | 27.30 | 36 | 2.67 |
| Lectures in other centers | 361 | 26.78 | 10 | 0.74 | 895 | 66.39 | 82 | 6.08 |
| Internet | 1033 | 76.63 | 76 | 5.64 | 205 | 15.21 | 34 | 2.52 |
| social media | 720 | 53.41 | 60 | 4.45 | 495 | 36.72 | 73 | 5.42 |
| Religious sources, e.g. the Church | 195 | 14.47 | 130 | 9.64 | 940 | 69.73 | 83 | 6.16 |
| n — the number of respondents; % — relative frequency | | | | | | | | |
